# Herbaceous peony *PlACLB2* positively regulates red petal formation by promoting anthocyanin accumulation

**DOI:** 10.3389/fpls.2022.992529

**Published:** 2022-09-28

**Authors:** Yuting Luan, Zijie Chen, Xin Wang, Hechen Zhang, Jun Tao, Daqiu Zhao

**Affiliations:** ^1^ College of Horticulture and Landscape Architecture, Yangzhou University, Yangzhou, China; ^2^ Institute of Horticulture, Henan Academy of Agricultural Sciences, Zhengzhou, China; ^3^ Joint International Research Laboratory of Agriculture and Agri-Product Safety, the Ministry of Education of China, Yangzhou University, Yangzhou, China

**Keywords:** *Paeonia lactiflora*, flower color, anthocyanin biosynthesis, ATP-citrate lyase, gene silencing

## Abstract

ATP-citrate lyase (*ACL*) gene catalyzes the formation of acetyl-CoA to provide intermediate precursors for many secondary metabolites, and also plays an important role in anthocyanin biosynthesis of plants. Herbaceous peony (*Paeonia lactiflora* Pall.) is an international cut flower known for its rich flower colors, however, the function of the *ACL* gene in flower color regulation is still unclear. Here, double-colored *P. lactiflora* ‘Hebao Jinlian’ were used to study the molecular mechanism of red petal, and acetyl-CoA and anthocyanin biosynthesis related *PlACLB2*, *PlCHS*, *PlDFR*, *PlANS*, and *PlbHLH1* genes were initially found to highly expressed in the red outer-petals. The expression pattern of *PlACLB2* was consistent with the spatial accumulation of anthocyanins. The correlation analysis of *PlACLB2* expression pattern, acetyl-CoA content, and anthocyanin accumulation revealed that *PlACLB2* was positively correlated with the acetyl-CoA and anthocyanin contents with correlation coefficients of 0.82 and 0.80. Moreover, multiple sequence alignment identified two typical conserved domains in PlACLB2, and phylogenetic analysis clustered PlACLB2 into the ACLB clade. PlACLB2 was localized in the nucleus and cytoplasm. On the one hand, silencing *PlACLB2* in *P. lactiflora* red outer-petal resulted in lighter petal color and decreased acetyl-CoA accumulation, and quantitative analysis detected that *PlACLB2*-silenced petals lost more anthocyanins than the control groups with a decrease of 31.0%, and the main pigment component cyanidin-3,5-*O*-diglucoside was reduced by 31.9%. On the other hand, overexpression of *PlACLB2* significantly promoted red coloration, acetyl-CoA content, and anthocyanin accumulation in tobacco flowers. These results demonstrated that *PlACLB2* promoted anthocyanin accumulation by increasing the abundance of its precursor substrate acetyl-CoA, thereby regulating the formation of the red petals in *P. lactiflora.*

## Introduction

ATP-citrate lyase (*ACL*) gene catalyzes the production of oxaloacetate and acetyl-CoA from citrate acid, which provides a precursor substrate for different metabolites including sugars, fatty acids, flavonoids, isoprenoids, etc. by manipulating carbon fluxes directions (Fatland et al., 2002; Fatland et al., 2005; Oliver et al., 2009; Chypre et al., 2012). In 1990, Elshourbagy et al. (1990) first cloned the *ACL* gene from *Rattus norregicus*, and then multiple *ACL* genes were isolated and characterized in a variety of plants, microorganisms, and animals, including *Lupinus albus*, *Arabidopsis thaliana*, *Saccharum sinense*, *Brassica napus*, *R. norregicus*, and *Rhodotorula glutinis* (Elshourbagy et al., 1990; Langlade et al., 2002; Fatland et al., 2002; Tong et al., 2009; Li et al., 2012; Lin et al., 2016). Over the past few decades, the functional studies of *ACL* genes have been mainly focused on animals and microorganisms (Hatzivassiliou et al., 2005; Migita et al., 2008; Meijer et al., 2009; Khwairakpam et al., 2015; Granchi, 2018), while the research on *ACL* genes in plants is still in its infancy. Like animals, *ACL* genes are also indispensable for plant growth, development, and abiotic stress response, because the catalytic product of *ACL*, acetyl-CoA, cannot be supplemented by other pathways, which has been proved to play key roles in many metabolic processes in plants (Fatland et al., 2005; Oliver et al., 2009; Xing et al., 2014). The *ACL* gene is a heterotetramer mainly present in the plant cytoplasm and consists of two subunits, *ACLA* and *ACLB* (Kim and Tabita, 2006). In *A. thaliana*, the *ACL* genes were composed of three ACLA subunits and two ACLB subunits (Fatland et al., 2002), whereas in *L. albus*, the *ACL* gene was encoded by only one ACLA subunit and one ACLB subunit. To date, the positive functions of *ACL* genes have been reported in the citrate acid accumulation of *Citrus reticulata*, drought resistance of *S. sinense*, and fatty acid biosynthesis of *B. napus* (Tong et al., 2009; Guo et al., 2020a; Zhu et al., 2021). In ornamental plants, only Zhao et al. (2020b) clarified the role of the *ACL* gene in balancing the overall metabolic homeostasis in *Petunia axillaris*. Apart from this, the report on the *ACL* gene in ornamental plants is almost blank and still needs to be further investigated.

Herbaceous peony (*Paeonia lactiflora* Pall.) is a traditional famous flower in China. It has been widely applied in the garden planting and potting due to its gorgeous appearance, and has been gradually developed as an emerging high-grade cut flower worldwide (Holloway and Buchholz, 2013; Yang et al., 2020b). The popularity of *P. lactiflora* is closely related to its rich flower colors divided into nine categories, including white, pink, red, purple, black, blue, green, yellow, and double-color (Wang and Zhang, 2005). Multiple pieces of research have revealed that flavonoids are the determining factor influencing *P. lactiflora* flower colors (Jia et al., 2008; Zhao et al., 2014; Zhao et al., 2016). At the physiological level, anthocyanins, dominated by cyanidin-based glycoside and peonidin-based glycoside confer *P. lactiflora* flowers with pink, red and purple performance (Zhao et al., 2012; Guo et al., 2019), as anthocyanins determine the flower color of more than 80% angiosperm families (Dai, 2010), while flavonols dominated by kaempferol-based glycoside and quercetin-based glycoside confer *P. lactiflora* flowers with white and yellow performance (Yang et al., 2020a). At the molecular level, the flavonoid biosynthesis pathway has been proved to be highly conserved both in model and non-model plants (Saito et al., 2013; Gao et al., 2021), as well as in ornamental plants such as *Gerbera hybrida*, Asiatic hybrid lily, *Freesia hybrida*, and *P. lactiflora* (Deng et al., 2014; Zhao et al., 2016; Yamagishi, 2018; Shan et al., 2020), and it was jointly regulated by the structural genes in this network. In *P. lactiflora*, the expression patterns of flavonoid-related genes have been studied in different cultivars with different flower colors including white, yellow, pink, and red, and their relationships have been preliminary clarified (Zhao et al., 2012; Zhao et al., 2016). In general, the high expression levels of the entire pathway genes or the downstream dihydroflavonol-4-reductase (*DFR*), anthocyanin synthase (*ANS*), and flavonoid 3-*O*-glucosyltransferase (*UFGT*) genes directed metabolism to anthocyanin accumulation to form pink, red or purple flowers, while the high expression of upstream chalcone synthase (*CHS*) and chalcone isomerase (*CHI*) genes promoted the formation of white and yellow flowers (Wu et al., 2018; Wu et al., 2022). Moreover, the microRNA-regulated flower color research has also been practiced in *P. lactiflora*, and a yellow color related miR156e-3p module has been identified (Zhao et al., 2017). Sun et al. (2020) found that the overexpression of *P. lactiflora PlDFR* and *PlANS* genes in *A. thaliana* and *Nicotiana tabacum* significantly increased the anthocyanin accumulation in leaves and flowers, while the functional studies of other flower color related genes in *P. lactiflora* have not been reported yet. However, most studies on *P. lactiflora* flower color formation were focused on the phenylalanine biosynthesis branch, while the research on the other branch, acetyl-CoA biosynthesis, is still blank and needs to be resolved.

In the previous study, the double-colored *P. lactiflora* cultivar ‘Hebao Jinlian’ was used to analyze the reasons for the specific pigmentation pattern of the red outer-petal, and anthocyanins were found to be the main factors contributing to its red outer-petals. At a deeper molecular level, it was found that the flavonoid-related structural genes also showed spatially differential expression patterns (Wang et al., 2022), while the function of *ACL* gene in flower color regulation has not been be clarified. Here, a candidate *ACL* gene was initially obtained based on comparative transcriptome analysis and its function in flower color regulation has been further studied by expression patterns analysis, subcellular localization observation, gene silencing, and heterologous transformation experiments. Taken together, it was the first time that the *ACL* gene was isolated from *P. lactiflora*, and it played a positive role in regulating the red pigmentation of *P. lactiflora* petals by promoting the acetyl-CoA and anthocyanin accumulation, which provided new insights into the study of *ACL* genes in flower color.

## Materials and methods

### Plant materials and growth conditions

The ground-planted double-colored *P. lactiflora* cultivar ‘Hebao Jinlian’, *Nicotiana benthamiana*, and *N. tabacum* ‘K326’ were used as plant materials. Petal samples of *P. lactiflora* at different developmental stages in May (S1, unfold-petal stage; S2, soft-bud stage; S3, initial-flowering stage; S4, full-flowering stage) were collected and partially stored at -80°C, which were prepared for next gene cloning, gene expression analysis, and virus-induced gene silencing (VIGS) assay. *N. benthamiana* and *N. tabacum* were grown in a greenhouse (25°C 16 h light/20°C 8 h dark), which were used for transient and stable transformation of candidate genes.

### RNA extraction and cDNA synthesis

Total RNA was extracted from different plant samples including *P. lactiflora* outer-petal and inner-petal and *N. tabacum* leaves by a MiniBEST Plant RNA Extraction Kit (TaKaRa, Japan) according to the manufacturer’s instruction. The quality control of RNA was performed by NanoDrop1000 spectrophotometry (Thermo Scientific, USA). For rapid-amplification of cDNA ends (RACE) cloning and quantitative real-time PCR (qRT-PCR) analysis, cDNA was synthesized from 1000 ng total RNA by a SMARTer RACE 5’/3’ Kit (Clontech, USA) and HiScript II Q RT SuperMix for qPCR (Vazyme, China).

### Gene cloning, bioinformatics analysis, multiple sequence alignment and phylogenetic analysis

The full-length cDNA of *PlACLB2* (cluster_11956) was isolated by RACE technology using SMARTer RACE 5’/3’ Kit (Clontech, USA) based on *P. lactiflora* ‘Hebao Jinlian’ full-length transcriptome database (NCBI sequence read archive ID: SRP257645). PCR products with predicted lengths of 5’, 3’, and full-length PCR amplification were purified and cloned into 5×TA/Blunt-Zero Cloning Mix vector (Vazyme, China) for sequence confirmation. The gene-specific primers are listed in **Supplementary Table S1**.

The coding sequence (CDS) and deduced amino acid sequence of PlACLB2 were obtained by BioXM 2.7.1 tools. The protein molecular formula, molecular weight, theoretical isoelectric point (pI), and instability coefficient of PlACLB2 were predicted by ProtParam (http://web.expasy.org/protparam/). The hydrophobic property of PlACLB2 was predicted by ProtScale (https://web.expasy.org/protscale/). The transmembrane structure and signal peptide of PlACLB2 were analyzed by TMHMM server 2.0 (http://www.cbs.dtu.dk/services/TMHMM) and SignalP 4.1 Server (http://www.cbs.dtu.dk/services/SignalP/). The secondary and tertiary structure predictions of PlACLB2 were conducted by SOPMA (https://npsa-prabi.ibcp.fr/cgi-bin/npsa_automat.pl?page=/NPSA/npsa_sopma.html) and SWISS MODEL (https://swissmodel.expasy.org/), respectively.

For sequence alignment, the full amino acid sequences of PlACL and ACL proteins from other plants were compared by DNAMAN. The conserved domains were highlighted with different colors. For phylogenetic analysis, the amino acid sequences of PlACL proteins from *P. lactiflora* and ACL proteins from other plants were aligned by the ClustalW and then subjected to MEGA 7.0 to generate a neighbor-joining tree.

### qRT-PCR analysis

*P. lactiflora* spatial (outer-petal and inner-petal) and temporal (S1 to S4) petals, gene-silenced petals, and gene-overexpressed *N. tabacum* petals were used to study the gene expression patterns. The cDNAs used for qRT-PCR were extracted and synthesized as mentioned above. Gene expression abundances were analyzed using NovoStart SYBR qPCR Super Mix (Novoprotein, China) by a BIO-RAD CFX Connect Optics Module (Bio-Rad, USA). The specific experimental details were referred to the previous study (Zhao et al., 2020a). The gene expression levels were normalized using *P. lactiflora Actin* (JN105299) and *N. tabacum Actin* (AB158612) as internal controls, respectively, and the final relative expression levels were calculated referring to the 2^-△△Ct^ method. All primers used are listed in **Supplementary Table S2**.

### Subcellular localization

The coding sequence of *PlACLB2* without stop codon was amplified by gene-specific primers (forward 5’-CGGGGATCCTCTAGAGTCGACATGGCGACCGGACAACTATTT-3’, reverse 5’-CACCATGGTACTAGTGTCGACCTTGGTGTAGAGAACATCTTCCCA-3’), and were fused upon the green fluorescent protein (GFP) N-terminal of the *p35S:GFP* vector (**Supplementary Figure S1A**). Next, the fusion constructs of *p35S:PlACLB2-GFP* and empty *p35S:GFP* were transformed into *Agrobacterium tumefaciens* strain *GV3101* by the freeze-thaw method. *Agrobacterium* cultures containing *p35S:PlACLB2-GFP* and empty *p35S:GFP* were used to inject 1-month-old *N. benthamiana* leaves. After 48 h, the GFP signals were observed by confocal laser microscopy (Nikon C2-ER, Japan) to determine the subcellular localization of PlACLB2.

### VIGS assay

The tobacco rattle virus (TRV)-based VIGS system composed of TRV1 and TRV2 vectors was applied to silence *PlACLB2* in *P. lactiflora* petals. The fragment of *PlACLB2* (forward 5’-AAGGTTACCGAATTCTCTAGACAAACGCAGCCTTCCTCGA-3’, reverse 5’-CGTGAGCTCGGTACCGGATCCTGGGTGACGGTATAGTGGCTG-3’) was first cloned into the TRV2 vector (**Supplementary Figure S1B**), and then the TRV1, TRV2, and TRV2-*PlACLB2* plasmids were transformed into *A. tumefaciens* strain *GV3101* as above. *Agrobacterium* cultures containing *PlACLB2*, TRV2, and TRV1 were mixed with a ratio of 1: 1 to infiltrate *P. lactiflora* petals at S1. The sterile water was used to remove the residual *Agrobacterium* liquid from the petals, then the petals were cultured on the 1/2 MS medium. After 5 d, the WT, TRV2, and *PlACLB2*-silenced petals were subjected to phenotype change observation, red representing a^*^ value determination, acetyl-CoA content and, total anthocyanin accumulation measurement, and gene expression level detection. All primers used are listed in **Supplementary Tables S2, S3**.

### Stable transformation in tobacco

The *p35S:PlACLB2-GFP* plasmids (**Supplementary Figure S1A**) were introduced for tobacco stable transformation. The fusion plasmids were transformed into *A. tumefaciens* strain *GV3101* as above. *Agrobacterium* cultures containing *PlACLB2* were transformed into tobacco ‘k326’ using the leaf disc method (Sunilkumar et al., 1999). T2 positive transgenic lines were used for the subsequent analysis. After ninety days of normal cultivation, PCR and qRT-PCR validation were used to identify *PlACLB2* transgenic plants. Meanwhile, the WT and transgenic lines at the full-flowering stage were subjected to flower phenotype change observation, red representing a^*^ value determination, acetyl-CoA content and total anthocyanin accumulation measurement, and gene expression level detection. All primers used are listed in **Supplementary Tables S2, S4**.

### Measurement of anthocyanins and acetyl-CoA

Total anthocyanin contents were quantified in both *P. lactiflora* and *N. tabacum* petals by high-performance liquid chromatography (HPLC) as previously described (Zhao et al., 2015). Briefly, 0.2 g *P. lactiflora* petals and 0.1 g tobacco flowers were added into 1.2 mL and 0.6 mL methanol solution (containing 70% methanol and 0.1% HCl) and were fully extracted in the dark for 24 h. The mixtures were centrifuged at 8,000 rpm for 10 min at 4°C to obtain the supernatant. Then, the supernatant was filtered through a 0.22 μm filter for the quantitative analysis using an Agilent 1200-6460 HPLC system (Agilent Technologies Inc., Santa Clara, USA), and cyanidin-3-*O*-glucoside was a standard for the semi-quantitative analysis of the total anthocyanin contents.

Acetyl-CoA content was measured using a plant acetyl-CoA ELISA kit (Shanghai Qiaodu Biotechnology Co., Ltd., China), and the specific operations were performed according to the instructions. Briefly, 0.1 g fresh petal samples were homogenized by vortexing with 0.5 M phosphate buffer (pH 7.4), and then the samples were centrifuged at 1,000 ×g for 20 mins to obtain the supernatant. For the enzyme-linked immunosorbent assay, the supernatant and acetyl-CoA antibody were kept in the microplate and incubated at 37°C for 1 h, After washing the microplate five times, the substrate was added and incubated at 37°C for 15 min. The acetyl-CoA content was determined by the SpectraMax M5 plate reader (Molecular Devices Corporation, Sunnyvale, CA, USA) by measuring absorbance at 450 nm, and the spectrophotometric values were substituted into the standard curve to calculate the melatonin contents.

### Statistical analysis

The variance of the results was analyzed with the SAS/STAT statistical analysis package (version 6.12, SAS Institute, Cary, NC, USA). Means were considered statistically significant at *P* < 0.05.

## Results

### *PlACLB2* might affect *P. lactiflora* petal anthocyanin biosynthesis

**Figure 1:**
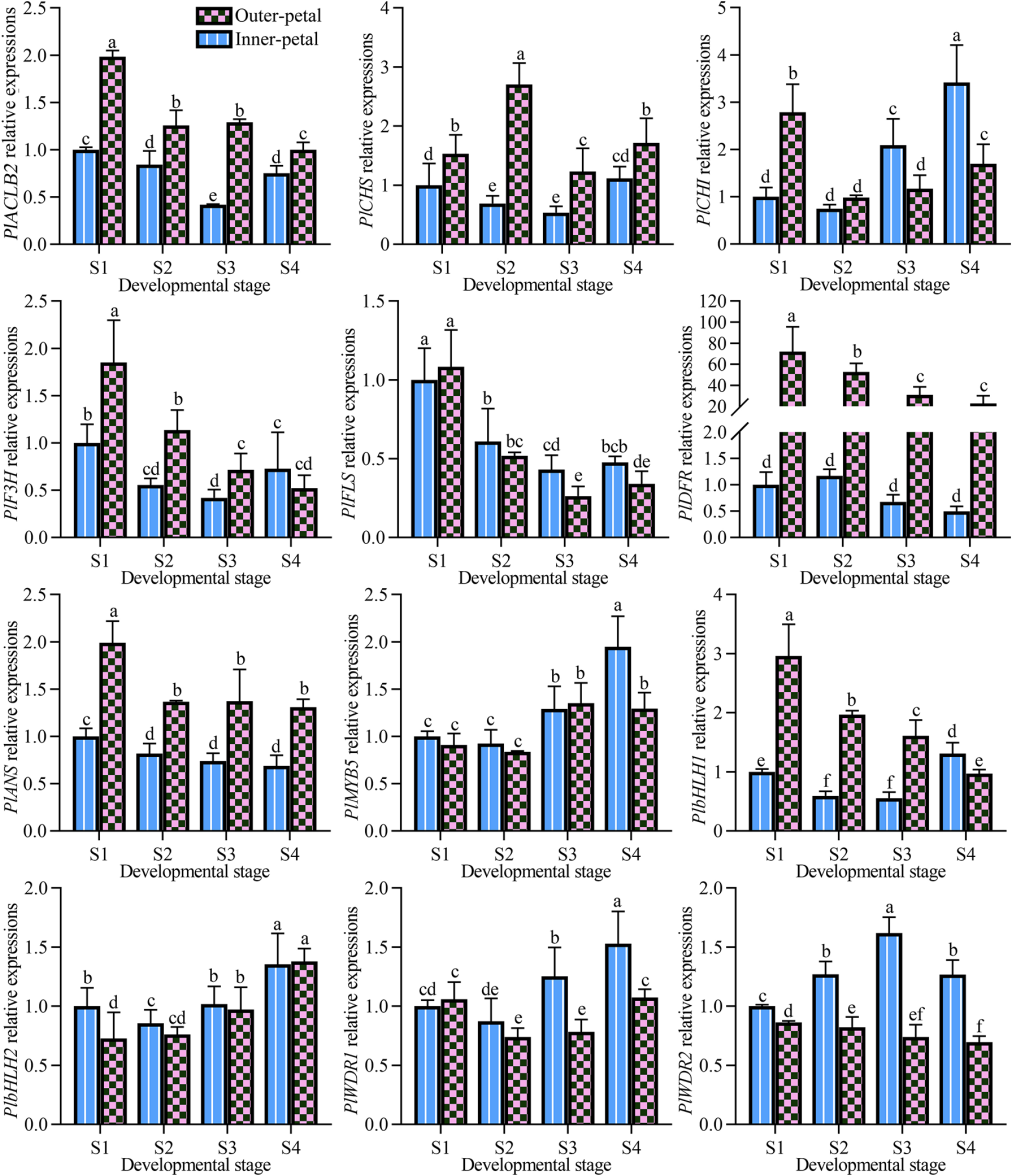
Expression pattern analysis of structural and regulatory genes related to anthocyanin biosynthesis pathway in *P. lactiflora* red outer-petals and yellow inner-petals from S1 to S4. S1, unfold-petal stage; S2, soft-bud stage; S3, initial-flowering stage; S4, full-flowering stage. The values represent the means ± SDs, and different letters indicate significant differences (*P* < 0.05).

In the previous study, the pigment accumulation of *P. lactiflora* ‘Hebao Jinlian’ petals was performed, and the outer-petal presented red due to the specific anthocyanin accumulation, while no anthocyanins accumulated in the yellow inner-petal (Wang et al., 2022). To further explore the molecular mechanism of anthocyanin biosynthesis in *P. lactiflora* ‘Hebao Jinlian’ outer-petal, the expression levels of structural and regulatory genes related to anthocyanin biosynthesis pathway were detected. As shown in **Figure 1**, the expression patterns of seven structural genes and five transcription factors were characterized both in outer-petals and inner-petals during four developmental stages. Among the structural genes, *PlACLB2*, *PlCHS*, *PlDFR*, and *PlANS* always demonstrated higher expression levels in outer-petals than in inner-petals, while other genes showed various expression patterns. The selected regulatory genes were the MBW homologous genes that regulated anthocyanin biosynthesis in *Arabidopsis thaliana.* Among them, only *PlbHLH1* was positively correlated with the accumulation of anthocyanins in *P. lactiflora*, and the other genes showed no difference or showed the opposite trends, which indicated that the regulatory genes regulating anthocyanin biosynthesis in *P. lactiflora* were not completely conserved in evolution and need to be further studied. At present, the research on *CHS*, *DFR* and, *ANS* genes is more in-depth, thus we mainly focused on the more upstream *ACL* gene, it was the catalytic enzyme of the acetyl-CoA precursor which provided the necessary substrate for anthocyanin biosynthesis.

**Figure 2:**
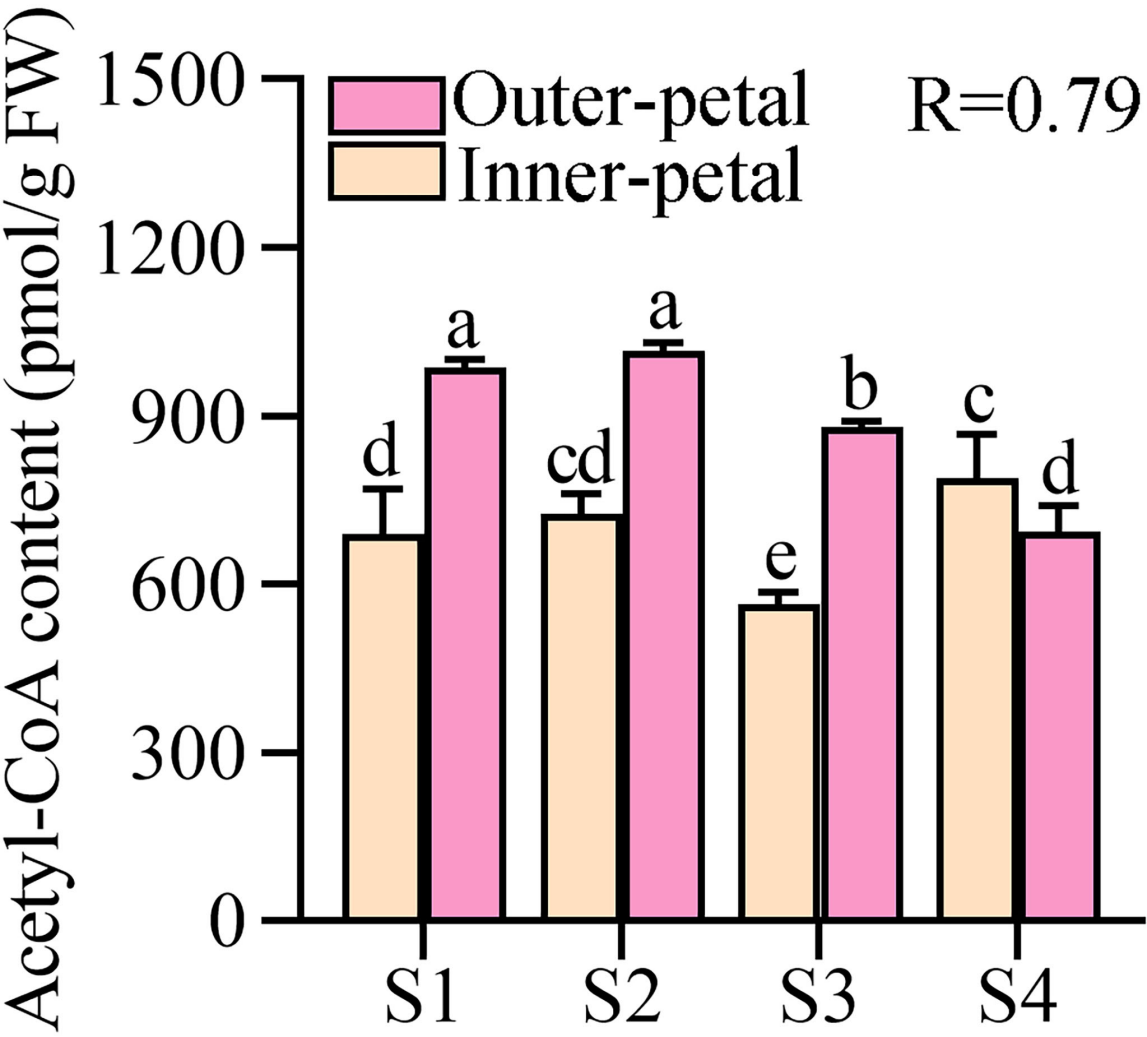
Quantitative analysis of acetyl-CoA in *P. lactiflora* red outer-petals and yellow inner-petals from S1 to S4. S1, unfold-petal stage; S2, soft-bud stage; S3, initial-flowering stage; S4, full-flowering stage. The values represent the means ± SDs, and different letters indicate significant differences (*P* < 0.05).

Next, we quantified the contents of acetyl-CoA in the outer-petal and inner-petal of ‘Hebao Jinlian’, and it was found that acetyl-CoA was highly accumulated in the outer petals at the first three developmental stages, and was 1.43, 1.40 and 1.56 times higher than in the inner-petals, respectively (**Figure 2**). Moreover, its content in the outer-petals decreased with the petal development, which was consistent with the changes of anthocyanin content with a high correlation coefficient (R) value of 0.79, which indicated that the content of acetyl-CoA in *P. lactiflora* petals was positively correlated with anthocyanin accumulation.

In *P. lactiflora* ‘Hebao Jinlian’, there exist three ACL subunits including two ACLA subunits and one ACLB subunit. *PlACLB2* was selected due to its relatively high expression levels in outer-petals. To further define whether there exists a relationship between *PlACLB2* gene and red pigmentation of *P. lactiflora* ‘Hebao Jinlian’ outer-petal, the spatial and temporal expression pattern of *PlACLB2* was detected in the outer-petals and inner-petals from S1 to S4. As shown in **Figure 1**, *PlACLB2* was always highly expressed in the outer-petal than in the inner-petal during the entire flower developmental stages. Notably, the expression level of *PlACLB2* reached the highest in the outer-petal at S1, and gradually decreased with the flower development with a total of 49.6% decrease. To deeply explore the function of *PlACLB2* gene in *P. lactiflora* ‘Hebao Jinlian’ outer-petal red pigmentation, the R values between *PlACLB2* expressions, acetyl-CoA content, and anthocyanin accumulation were calculated, and *PlACLB2* expressions showed 0.82 and 080 R-value with acetyl-CoA content and anthocyanin content. These results indicated that *PlACLB2* positively affect the anthocyanin biosynthesis in *P. lactiflora* petals by controlling acetyl-CoA biosynthesis.

### Isolation and characterization of *PlACLB2*

**Figure 3:**
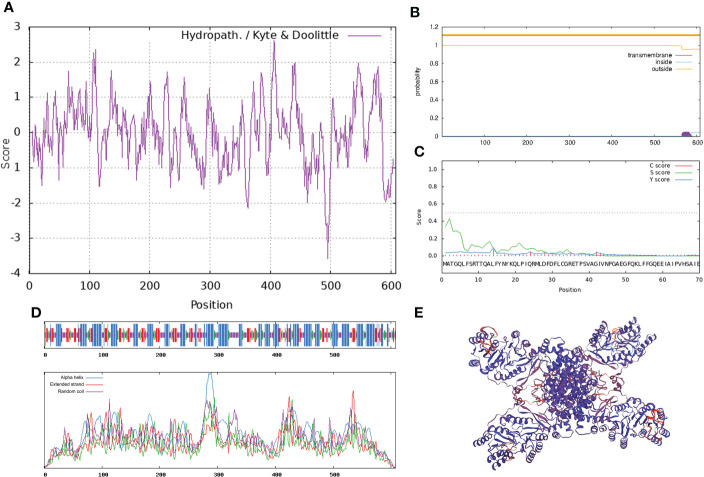
Bioinformatics analysi**s** of PlACLB2. **(A)** The hydrophobic property prediction of PlACLB2. **(B)** The transmembrane structure prediction of PlACLB2. **(C)** The signal peptide prediction of PlACLB2. **(D)** The secondary structure prediction of PlACLB2. **(E)** The tertiary structure prediction of PlACLB2.

Combining the results of *P. lactiflora* ‘Hebao Jinlian’ full-length transcriptome database and RACE technology, the full-length sequence of *PlACLB2* was obtained, and it was 2,147 bp in length (134 bp 5’ non-coding region; 186 bp 3’ non-coding region), which encoded 608 amino acids (GenBank accession number: ON960073). ProtParam online software showed that the molecular formula of PlACLB2 protein was C_2961_H_4679_N_797_O_866_S_22_; its molecular weight was 66.00 kD, and the pI was 7.59. The instability coefficient of PlACLB2 protein was 30.99, indicating that PlACLB2 was a stable protein. ProtScale identified PlACLB2 as a hydrophilic protein (**Figure 3A**). TMHMM server 2.0 and SignalP 4.1 Server showed that there did not exist transmembrane structure and signal peptide site in the PlACLB2 protein (**Figures 3B, C**). SOPMA analysis predicted that the secondary structure of PlACLB2 protein was mainly composed of alpha-helix (247, 40.62%), extended strand (108, 17.76%), and random coil (189, 31.09%) (**Figure 3D**). SWISS MODEL was used to homogenously mimic the tertiary structure of PlACLB2 protein, and 53.72% sequence identify was detected between PlACLB2 and human ACL proteins (**Figure 3E**).

**Figure 4:**
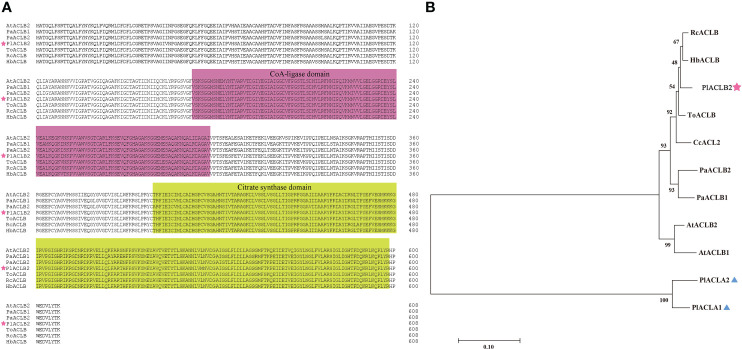
Amino acid sequence alignment and phylogenetic analysis of PlACLB2 and other ACL proteins. **(A)** Multiple sequence alignment of the amino acid sequence of PlACLB2 with ACL proteins from other plants. PlACLB2 isolated from *P. lactiflora* was marked with a pentagram. The distinct parts indicate conserved CoA-ligase and citrate synthase domains. **(B)** A phylogenetic tree of PlACLB2 and other ACL proteins in other plants. PlACLB2 was indicated with a pentagram. All these protein sequences were downloaded from NCBI and their GenBank IDs are as follows: HbACLB (XP_021653557.1, *Hevea brasiliensis*), RcACLB (XP_015574748.1, *Ricinus communis*), ToACLB (PON93182.1, *Trema orientale*), CcACL2 (XP_006419381, *Citrus clementina*), PaACLB1 (Peaxi162Scf00228g00327.1, *Petunia axillaris*), PaACLB2 (Peaxi162Scf01160g00016.1, *Petunia axillaris*), AtACLB1 (NP_001326324.1, *Arabidopsis thaliana*), AtACLB2 (NP_001332247.1, *Arabidopsis thaliana*).

By comparing PlACLB2 with ACL proteins from *A. thaliana*, *P. axillaris*, *Trema orientale*, *Ricinus communis*, and *Hevea brasiliensis*, it was found that PlACLB2 had typical ACL family domains, including a CoA-ligase domain from 173 to 298 and a citrate synthase domain from 400 to 598. Moreover, the ACL proteins were highly conserved among different species, and the sequence similarity was always higher than 96% (**Figure 4A**). Moreover, the PlACLA1 and PlACLA2 proteins were also highly conserved in evolution, and multiple sequence alignment indicated that they had two typical ACL family domains, including a ATP-grasp domain from 5 to 204 and a ATP citrate lyase citrate-binding domain from 241 to 418 (**Supplementary Figure S2**). To define PlACLB2, the phylogenetic tree analysis of PlACLA1, PlACLA2, PlACLB2 and, eight ACL proteins from *H. brasiliensis*, *R. communis*, *T. orientale*, *Citrus clementina*, *P. axillaris*, and *A. thaliana* was performed. As result, PlACLB2 and PlACLAs were divided into two separate branches, and PlACLB2 was identified as highly homologous to ACLB proteins in other plants, so it was initially named PlACLB2 (**Figure 4B**).

**Figure 5:**
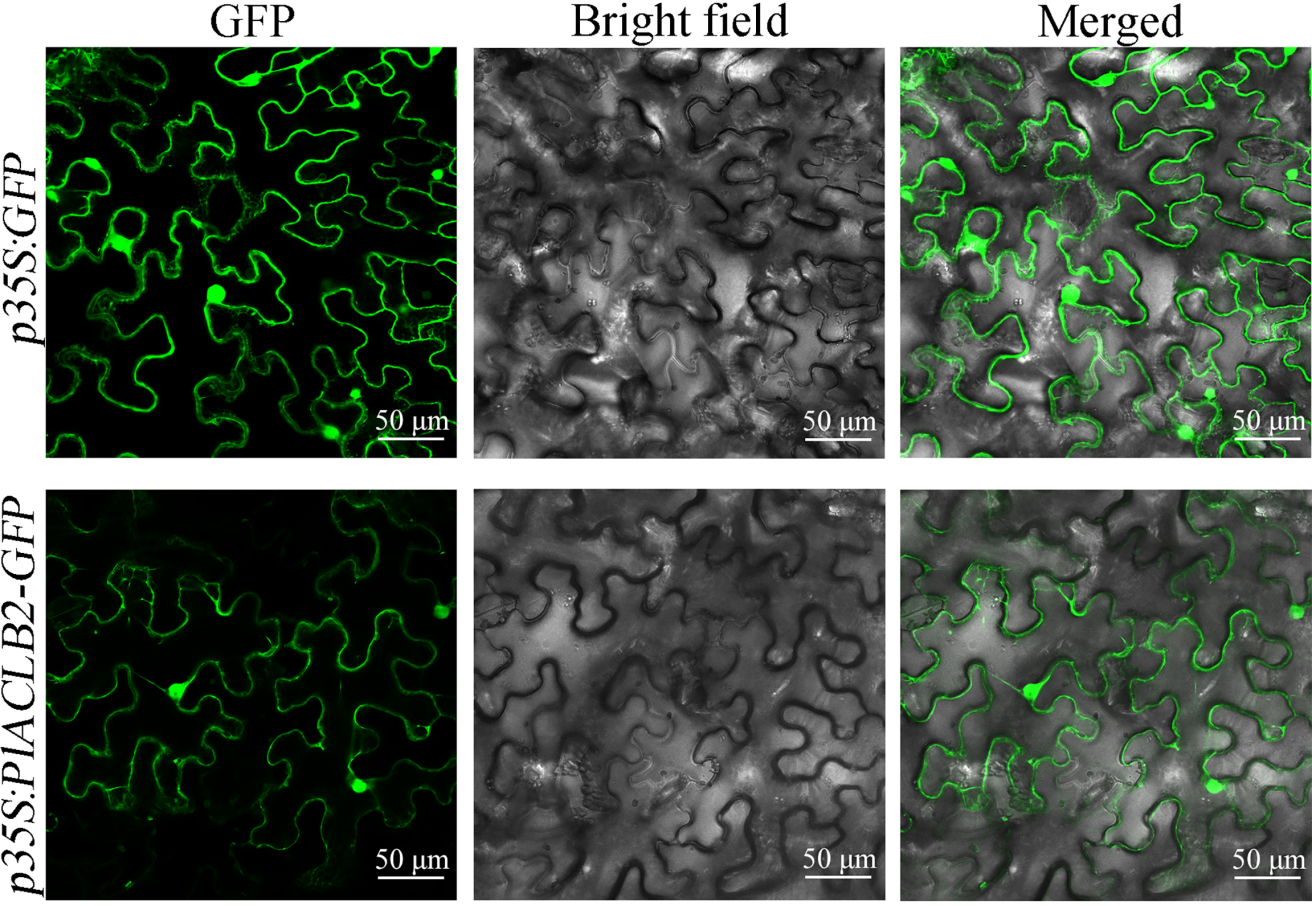
Subcellular localization of PlACLB2 in tobacco leaves. The green fluorescences driven by *p35S:GFP* and *p35S:PlACLB2-GFP* were visualized at 488 nm wavelength.

The subcellular localization of PlACLB2 was observed *via* tobacco leaf epidermal cells, and the GFP signals driven by *p35S:PlACLB2-GFP* vector were detected both in the nucleus and cytoplasm, indicating PlACLB2 was located in the nucleus and cytoplasm in tobacco system (**Figure 5**).

### Silencing *PlACLB2* inhibits red pigmentation in *P. lactiflora* petals

**Figure 6:**
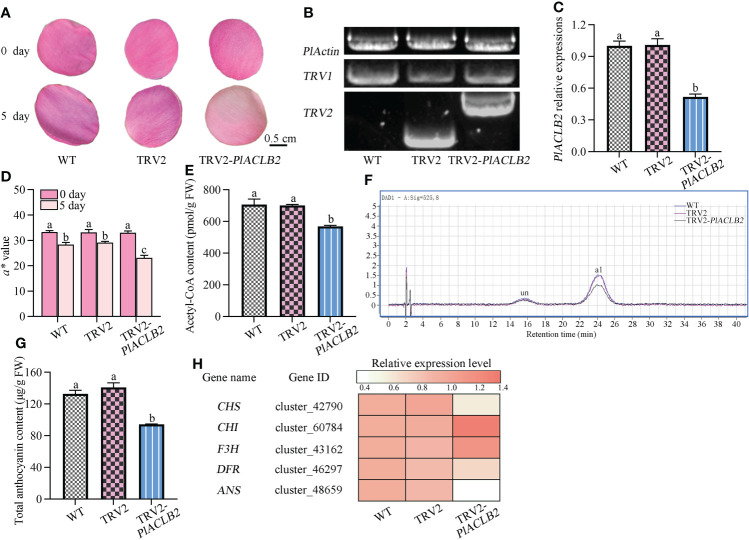
VIGS of *PlACLB2* in *P. lactiflora* outer-petals. **(A)** Flower phenotype changes of the WT, empty vector, and *PlACLB2*-silenced petals. **(B)** PCR validation of *PlACLB2* transgenic petals. **(C)** qRT-PCR validation of *PlACLB2* transgenic petals. **(D)** Measurement of red representing a^*^ value in the WT, empty vector, and *PlACLB2* transgenic petals at 0 day and 5 day. **(E)** Measurement of acetyl-CoA content in the WT, empty vector, and *PlACLB2* transgenic petals at 5 day. **(F)** HPLC analysis of anthocyanin accumulation in the WT, empty vector, and *PlACLB2* transgenic petals at 5 day. **(G)** Measurement of total anthocyanin content in the WT, empty vector, and *PlACLB2* transgenic petals at 5 day. WT, wild type; un, unidentified; a1, cyanidin-3,5-*O*-diglucoside. **(H)** Heat map of expression patterns of anthocyanin biosynthesis related genes in the WT, empty vector, and *PlACLB2* transgenic petals at 5 day. The values represent the means ± SDs, and different letters indicate significant differences (*P* < 0.05).

The TRV-based transient transformation system was used to study the function of *PlACLB2* in anthocyanin accumulation due to their lack of a genetic transformation system in *P. lactiflora*, and the outer-petal at S1 was used as plant materials. The a^*^ value was used as the basis for choosing similar petals, and each treatment included at least thirty biological replicates. Five days after infection, the phenotype changes were observed between the wild type (WT), TRV2,and *PlACLB2*-silenced petals, and it was found that silencing *PlACLB2* resulted in a more loss of red pigments when compared with the WT and TRV2 groups (**Figure 6A**). Moreover, PCR and qRT-PCR proved that the TRV2-silencing vector was successfully transformed into the *P. lactiflora* petals, and the mRNA level of *PlACLB2* was significantly inhibited by a decrease of 48.3% (**Figures 6B, C**). By measuring the a^*^ value at 5 day of different groups, it was found that the a^*^ value of *PlACLB2*-silenced petals was much lower than the WT and TRV2 groups with a decrease of 18.3% and 20.5%, which matched the phenotype changes (**Figure 6D**). Moreover, the acetyl-CoA content in *PlACLB2*-silenced petals decreased by an average of 19.23% (**Figure 6E**). Next, the anthocyanin accumulations were determined, and HPLC detected two anthocyanins as previously reported (Wang et al., 2022), and then the quantitative analysis indicated that *PlACLB2*-silenced petals lost more anthocyanins, which accounted for an average of 31.0% decrease than the WT and TRV2 groups, and the main pigment component cyanidin-3,5-*O*-diglucoside (Cy3G5G) was reduced by 31.9% (**Figures 6F, G**). In addition, silencing *PlACLB2* also down-regulated the expression levels of anthocyanin-related structural genes *CHS*, *DFR*, and *ANS* in *P. lactiflora* (**Figure 6H**). These results indicated that *PlACLB2* functioned positively in *P. lactiflora* petal acetyl-CoA and anthocyanin biosynthesis and then contributed to the formation of red petals.

### Overexpression of *PlACLB2* promotes anthocyanin accumulation in transgenic tobacco flowers

**Figure 7:**
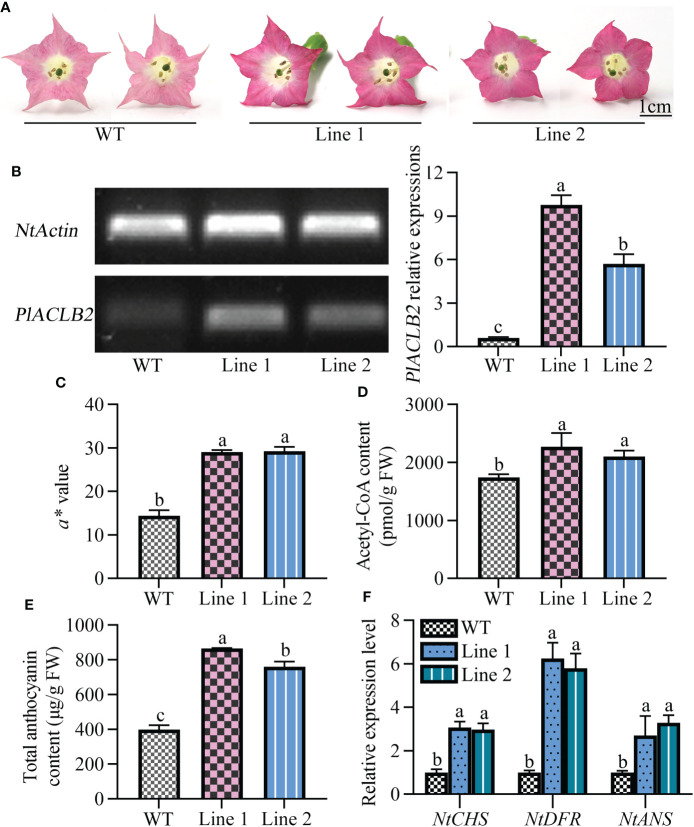
Stable transformation of *PlACLB2* in tobaccos. **(A)** Flower phenotype changes of the WT and *PlACLB2* transgenic tobaccos. **(B)** Expression analysis of *PlACLB2* in the WT and *PlACLB2* transgenic tobaccos validated by PCR and qRT-PCR. **(C)** Measurement of red representing a^*^ value and total anthocyanin content in the WT and *PlACLB2* transgenic tobaccos. **(D)** Measurement of acetyl-CoA content in the WT and *PlACLB2* transgenic tobaccos. **(E)** Measurement of total anthocyanin content in the WT and *PlACLB2* transgenic tobaccos. **(F)** Expression levels of anthocyanin biosynthesis related genes in the WT and *PlACLB2* transgenic tobaccos. WT, wild type. The values represent the means ± SDs, and different letters indicate significant differences (*P* < 0.05).

To further characterize the function of *PlACLB2* in *P. lactiflora* red petal pigmentation, the stable transformation of *PlACLB2* was performed in tobaccos. As shown in **Figure 7A**, the flowers of *PlACLB2* overexpression tobaccos at the full-flowering stage were much redder than the WT, which meant that the red flower phenotype has been stably inherited in the *PlACLB2* transgenic tobaccos. Moreover, PCR and qRT-PCR were applied to detect the presence and expression level of *PlACLB2* in tobaccos, and *PlACLB2* was strongly expressed in the two transgenic lines, with an average of 12.9-fold than in WT (**Figure 7B**). Then, *PlACLB2* transgenic tobaccos were subjected to flower color indices measurement, and both of the two transgenic lines demonstrated much higher a^*^ value, with an average of 2-fold than in WT, which further confirm the visual conclusion of redder flowers (**Figure 7C**). In addition, enzyme-linked immunosorbent and HPLC assays were also used to quantitative the acetyl-CoA and anthocyanin accumulations in tobacco flowers, and the acetyl-CoA and total anthocyanin contents in *PlACLB2* transgenic tobacco flowers increased by an average of 25.35% and 103.15%, respectively (**Figures 7D, E**). Also, the expression levels of *CHS*, *DFR*, and *ANS* genes in *PlACLB2* transgenic tobacco significantly increased when compared with the WT (**Figures 7F**). These results indicated that overexpression of *PlACLB2* in tobacco increased acetyl-CoA and anthocyanin accumulations and led to the redder phenotype of flower color.

## Discussion

As an important cytoplasmic enzyme, ACL is the rate-limiting enzyme of various metabolic pathways in plants, and its catalytic product, acetyl-CoA, is the precursor of the flavonoid biosynthesis pathway, which further affects the biosynthesis of the downstream anthocyanins (Xing et al., 2014; Li et al., 2017). In *P. lactiflora*, anthocyanins contribute to rich flower color changes, including double-color rendering patterns (Zhao and Tao, 2015), but its research is still limited to the basic level and needs to be further developed. In this study, we explored the role of the *ACL* gene in the anthocyanins biosynthesis of the double-colored *P. lactiflora* cultivar ‘Hebao Jinlian’ to discuss the underlying molecular mechanism of the red outer-petal formation.

The red petals of *P. lactiflora* were attributed to the specific accumulation of anthocyanins, and the structural genes influencing its biosynthesis have been isolated from *P. lactiflora* with different flower colors. For instance, the upstream *PlPAL*, *PlF3’H*, and *PlF3H* genes were highly expressed in the red-colored cultivars ‘Dafugui’ and ‘Dahonglou’, which provided sufficient substrates for downstream anthocyanins biosynthesis (Zhao et al., 2016; Wu et al., 2022). While in the cultivar ‘coral sunset’ (change from coral to yellow), the expression level of the upstream *PlCHS* gene at the pigmented stage was 1,500 times higher than that at the flowering-wilting stage, and it was the most differentially expressed structural gene in the entire biosynthesis pathway (Guo et al., 2019). In Japanese gentian, the post-transcriptional silencing of the *CHS* gene resulted in blue-white double-colored corolla (Ohta et al., 2022), while in *Camellia sinensis*, *CsPAL4* was identified to be positively related to anthocyanin accumulation in purple-leaf tea by correlation analysis (Chen et al., 2022). All the above evidence indicates that the anthocyanin accumulation in plant organs is closely related to the expression abundance of upstream genes, and this specificity seems to be more significant in *P. lactiflora* flowers than in other plants. In the anthocyanin biosynthesis pathway, the *ACL* gene is located further upstream of the *CHS* gene, at the other branch as the *PAL* gene, which also provides necessary substrates for anthocyanin biosynthesis, but few studies focused on it. In our previous study, comparative transcriptome analysis was used to explore the key genes that distinguish the red outer-petal from the yellow inner-petal (NCBI sequence read archive ID: SRP257895). Here, the acetyl-CoA content in ‘Hebao Jinlian’ outer-petals and inner-petals was quantified and a candidate *ACL* gene was found. Next, the expression pattern analysis showed that *PlACLB2* was always highly expressed in the red outer-petal and was consistent with the flower color changes, and its expression levels had high correlation coefficients with the acetyl-CoA and anthocyanin content in petals. Multiple sequence alignment showed that it had two typical ACL gene conserved domains as ACL members in other plants. It was evolutionarily clustered into the ACLB clade and initially named *PlACLB2*. In *Citrus grandis*, *CitACLB1* was highly expressed in mature leaves than in juice sacs, which accumulated more starch, flavonoid, and carotenoid (Guo et al., 2020b). The blood oranges under cold storage accumulated more anthocyanins and flavonoids, which might be attributed to the fact that low-temperature-induced the upregulation of *ACL* gene expressions (Crifò et al., 2011). PlACLB2 was located both in the nucleus and cytoplasm, which was consistent with *C. grandis CitACLA1*, *CitACLA2*, and *CitACLB1* (Guo, 2020). These findings indicated that PlACLB2 functioned as a typical cytoplasmic enzyme and might be responsible for the red outer-petal formation in *P. lactiflora* by providing enough acetyl-CoA for anthocyanin biosynthesis.

To verify whether *PlACLB2* influences the anthocyanin biosynthesis in *P. lactiflora*, the VIGS experiment was applied to test its function by silencing its mRNA level expressions considering that there does not exist a genetic transformation system in *P. lactiflora*. Five days after infection, *PlACLB2*-silenced petals demonstrated much a lighter petal color when compared with the control groups, it was initially attributed to the fact that 48.3% of expressions of *PlACLB2* were blocked by the VIGS technology. Furthermore, the redness representing a^*^ value was evaluated at 0 and 5 days in all groups, and the *PlACLB2*-silenced petals showed a larger drop. After deeply analyzing the acetyl-CoA and anthocyanin accumulation in petals, we found that the *PlACLB2*-silenced petals accumulated less acetyl-CoA and anthocyanin when compared with the WT and TRV2 groups, which accounted for an average of 19.23% and 31.0% decrease, and the main anthocyanin component Cy3G5G was reduced by 31.9%. These results might be attributed to the fact that silencing *PlACLB2* also inhibited the expression levels of the downstream *PlCHS*, *PlDFR*, and *PlANS* genes. In *P. axillaris* flowers, silencing *PaACL* family members all resulted in the reduction of total anthocyanin contents, which was consistent with the role of *PlACLB2*. These results suggested that *PlACLB2* acted as a positive anthocyanin regulator in *P. lactiflora* red outer-petal formation.

Then, the function of *PlACLB2* was further verified through heterologous overexpression in tobaccos. After positive validation of the transgenic tobaccos, the flowers at the full-flowering stage were subjected to phenotype change observation, and obvious red pigmentation was observed in *PlACLB2* transgenic tobaccos compared with the WT. Meanwhile, overexpression of *PlACLB2* in tobaccos not only increased the redness representing a^*^ value (2-fold) but also accumulated more acetyl-CoA and anthocyanin in the flowers (an average of 25.35% and 103.15%). Meanwhile, the anthocyanin biosynthesis related *NtCHS*, *NtDFR*, and *NtANS* genes also demonstrated higher expression levels when compared with the WT. In transgenic *C. grandis* callus, the content of flavonoids was significantly increased when overexpressing the *CitACLB1* gene (Guo, 2020). In this study, overexpression of *P. lactiflora PlACLB2* promoted anthocyanin accumulation in tobaccos, and it was the first time that the positive function of the *ACL* gene in anthocyanin biosynthesis was verified by a heterologous system. In *C. grandis* fruits, an upstream transcription factor CitERF6 decreased citric acid content by upregulating *CitACLA1* expressions, and whether there exist upstream regulatory genes regulating *P. lactiflora PlACLB2* expressions needs to be further investigated.

## Conclusion

In the present study, the first *ACL* family member *PlACLB2* was isolated from *P. lactiflora* petals based on its putative function in regulating anthocyanin biosynthesis. *PlACLB2* was highly expressed in the red outer-petals that accumulated more acetyl-CoA and anthocyanin than the yellow inner-petals, and showed a positive trend with the spatial and temporal anthocyanin changes in *P. lactiflora* petals, and it was defined as a typical ACLB subunit with the nucleus and cytoplasm expressions. Subsequently, silencing and overexpression of *PlACLB2* resulted in loss and more accumulation of total anthocyanins in *P. lactiflora* and tobacco flowers, which further confirmed the positive role of *PlACLB2* in acetyl-CoA and anthocyanin accumulation and red outer-petal formation. Overall, it was the first time that the *ACL* family member was isolated from *P. lactiflora*, and these results provided a reference for the flower color study of *ACL* genes in *P. lactiflora* as well as in other ornamental plants.

## Data availability statement

The data presented in the study are deposited in the NCBI repository, accession number SRP257645 and SRP257895.

## Author contributions

DZ and JT conceived and designed the project. YL, ZC and XW performed the experiments. YL, ZC, XW, HZ, DZ and JT participated in discussions and contributed to the writing of the article. All authors contributed to the article and approved the submitted version.

## Funding

This work was supported by the National Key R&D Program of China (2018YFD1000405), Modern Agriculture (Flower) Industrial Technology System of Jiangsu Province (JATS[2022]489), Forestry Science and Technology Prossmotion Project of Jiangsu Province (LYKJ[2021]01) the Key Disciplines of Jiangsu Province, Postgraduate Research and Practice Innovation Program of Jiangsu Province (KYCX22_3521), Qing Lan Project of Jiangsu Province and High-Level Talent Support Program of Yangzhou University.

## Conflict of interest

The authors declare that the research was conducted in the absence of any commercial or financial relationships that could be construed as a potential conflict of interest.

## Publisher’s note

All claims expressed in this article are solely those of the authors and do not necessarily represent those of their affiliated organizations, or those of the publisher, the editors and the reviewers. Any product that may be evaluated in this article, or claim that may be made by its manufacturer, is not guaranteed or endorsed by the publisher.
