# Supplementary material for: Herbaceous peony PlACLB2 positively regulates red petal formation by promoting anthocyanin accumulation
Source: Front Plant Sci. 2022 Sep 28;13:992529. doi: 10.3389/fpls.2022.992529 (PMC9554437; doi:10.3389/fpls.2022.992529)
Supplement: Supplementary file 1 [file DataSheet_1.doc]

**Supplementary Table S1** Gene-specific primers used for 5’ /3’ RACE and full-length PCR amplification of *PlACLB2*.

| Gene ID | Primer | Sequence (5' - 3') | Application |
| --- | --- | --- | --- |
| cluster_11956 | *PlACLB2*_1_ | AAGCAAAGAATCAAGCACTG | 1^st^ of 3′ RACE |
|  | *PlACLB2*_2_ | AGCAAGGGCAGGAAAGGA | 2^nd^ of 3′ RACE |
|  | *PlACLB2*_3_ | TATGCCCAATGAGACCAATGGAACGG | 5′ RACE (Clontech) |
|  | *PlACLB2*_4_ | FP: CAACGCAGAGTGAGTCTT | full-length PCR amplification |
|  |  | RP: CTTGTCAAAAGCAGGTAC |  |

FP, forward primer; RP, reverse primer.

**Supplementary Table S2** Gene-specific primers used for qRT-PCR detection in this study.

| Gene name | Gene ID | Forward primer (5' - 3') | Reverse primer (5' - 3') |
| --- | --- | --- | --- |
| *PlActin* | JN105299 | GTTGCCCTTGATTACGAG | CAGCTTCCATTCCGATTA |
| *NtActin* | AB158612 | TCCTCATGCAATTCTTCG | ACCTGCCCATCTGGTAAC |
| *PlACLB2* | cluster_11956 | TGGTGGACGAGATGAGT | CAGTGCTTGATTCTTTGC |
| *PlCHS* | cluster_42790 | ACCCCTGCTCATTGTATC | ATGGTGCCATGTATTCAC |
| *PlCHI* | cluster_60784 | CATCGGCGTCTACTTGGA | ACGGCAGGATCATTGTCA |
| *PlF3H* | cluster_43162 | TAGTGACCTACTTTTCATACCC | CGTTTGTGAGTGCTTCCT |
| *PlFLS* | cluster_56207 | ACACTGTTGAAGGGTATGG | TAGTTGACGCAAGAAGGC |
| *PlDFR* | cluster_46297 | GAGTCGTTTTCACATCATC | CATCCATCCAGTCATCTT |
| *PlANS* | cluster_48659 | CAAACCAGCATCACCAAC | CTTTCCAAAAGCTCATCAGA |
| *PlMYB5* | cluster_46884 | GCTGGCTTACTCCGATGT | AAAGACCACCGATTTCCC |
| *PlbHLH1* | cluster_6268 | CAATGGGGCAATAAAGAC | ATTCCGTTAAGTCTTCCG |
| *PlbHLH2* | cluster_8628 | GTAGTATGTTTTCCCCACC | GAGGAAGATTTCTCGGAG |
| *PlWDR1* | cluster_55091 | TATCTACGCCTCTGGGAA | AAGTGGTGTCAATGCTCG |
| *PlWDR2* | cluster_41855 | CGCTACTTCCAGTGATTT | ACCTATACGCTTCGGCTC |
| *NtCHS* | NM_001326166.1 | ACTGCGGTCACATTTCGT | ATCCGGGAGTAGGGTTTG |
| *NtDFR* | NM_001325630.2 | CGAGGACCCTGAGAATGA | CAGCCGATGAAGTGAAAA |
| *NtANS* | XM_016598316.1 | GCCAAACAGATTAGGAAC | TCATTTGAAGCAGTAGGTC |

**Supplementary Table S3** Gene-specific primers used for *P. lactiflora* line identification.

| Gene name | Gene ID | Forward primer (5' - 3') | Reverse primer (5' - 3') | Application |
| --- | --- | --- | --- | --- |
| *PlActin* | JN105299 | GTTGCCCTTGATTACGAG | CAGCTTCCATTCCGATTA | PCR identification |
| *TRV1* | / | ACTAACCTGGGCGAAGGACAC | CGGACTCAGATGCCGAATACA | PCR identification |
| *PlACLB2* | cluster_11956 | TTGTTACTCAAGGAAGCACGAT | TCCCCTATGGTAAGACAATGAG | PCR identification |

**Supplementary Table S4** Gene-specific primers used for transgenic tobaccos lines identification.

| Gene name | Gene ID | Forward primer (5' - 3') | Reverse primer (5' - 3') |
| --- | --- | --- | --- |
| *NtActin* | AB158612 | TCCTCATGCAATTCTTCG | ACCTGCCCATCTGGTAAC |
| *PlACLB2* | cluster_11956 | TGGTGGACGAGATGAGT | CAGTGCTTGATTCTTTGC |

**
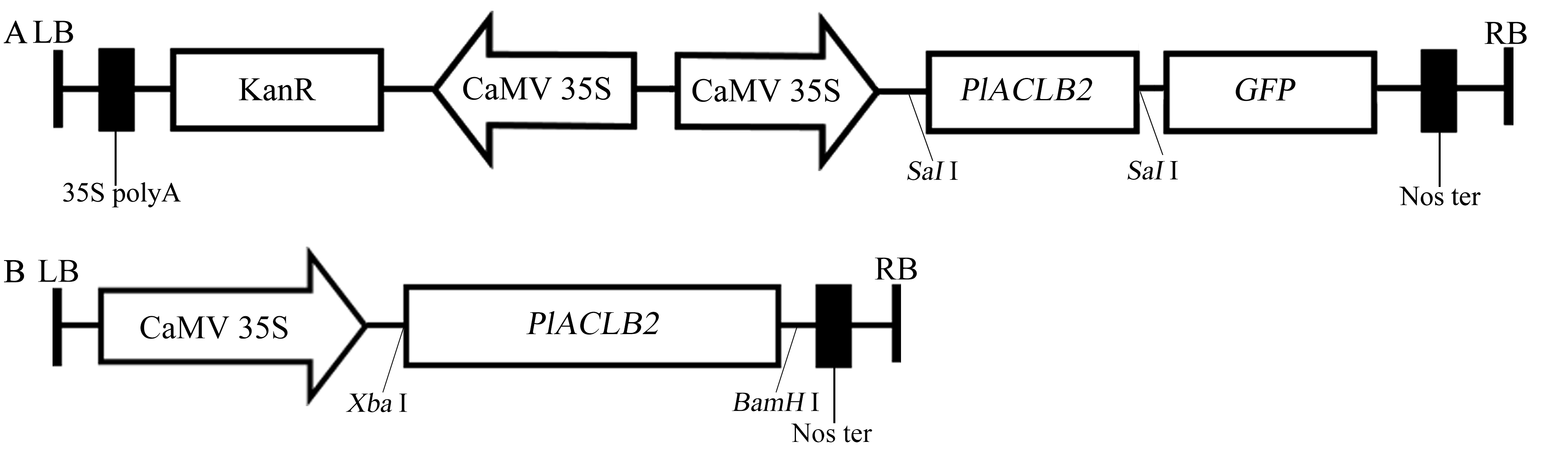
**

**Supplementary Figure S1** The plasmid profile of *PlACLB2* overexpression and VIGS vectors promoted by CaMV35S promoter for transient and stable transformation.

**

Supplementary Figure S2** Multiple sequence alignment of the amino acid sequence of PlACLAs with ACL proteins from other plants. PlACLAs from *P. lactiflora* were marked with triangles. The distinct parts indicate conserved ATP-grasp and ATP citrate lyase citrate-binding domains. All these protein sequences were downloaded from NCBI and their GenBank IDs are as follows: CitACLA1 (XP_006483902, *Citrus sinensis*), CitACLA2 (XP_006480297, *Citrus sinensis*), PaACLA1 (Peaxi162Scf00357g00428.1, *Petunia axillaris*), PaACLA2 (Peaxi162Scf00158g00011.1, *Petunia axillaris*), AtACLA1 (AT1G10670, *Arabidopsis thaliana*), AtACLA2 (AT1G60810, *Arabidopsis thaliana*), AtACLA3 (AT1G09430, *Arabidopsis thaliana*).
